# Rowers’ Self-Reported Behaviors, Attitudes, and Safety Concerns Related to Exercise, Training, and Competition During Pregnancy

**DOI:** 10.7759/cureus.1534

**Published:** 2017-08-01

**Authors:** Ashley Franklin, Joanna Mishtal, Teresa Johnson, Judith Simms-Cendan

**Affiliations:** 1 Alaska Family Medicine Residency; 2 Department of Anthropology, University of Central Florida; 3 Office of Assessment and Evaluation, Johns Hopkins University School of Medicine; 4 University of Central Florida College of Medicine

**Keywords:** exercise during pregnancy, pregnant athlete, rower, crew, antenatal exercise, exercise, pregnancy, rowing, sports medicine, preventive medicine

## Abstract

Background

The American College of Obstetrics and Gynecology notes that pregnant athletes require more supervision due to their involvement in strenuous training schedules throughout pregnancy. Currently, rowing is not mentioned in the guidelines despite its increasing popularity, high cardiovascular demands, and risk for abdominal trauma.

Methods

This study aimed to elicit information from competitive female rowers regarding exercise, training, and competition during pregnancy. We administered a survey consisting of 122 items to female Masters rowers in the United States, aged 21 to 49 years, from June to December 2013.

Results

A total of 224 recreational and elite rowers met the inclusion criteria. Pregnant rowers self-reported high levels of exercise engagement: 85.2% (n/N = 98/115) exercised during any past pregnancy; exercise adherence decreased throughout pregnancy with 51.3%, 42.4%, and 15.7% meeting and/or exceeding national guidelines during the first, second, and third trimesters, respectively. Rowers were significantly (p < 0.001) more likely to state that an activity at a specified intensity and trimester was unsafe if they were younger, had less rowing experience, or were nulliparous. Decreased perceived rowing safety was associated with on-water training, higher intensity exercise, competition, and increasing gestational age. Primary safety concerns were the risk of oar-induced abdominal trauma and physiological effects due to high intensities required by the sport. Novel barriers to exercise in pregnancy included guilt towards the team and a mental barrier due to decreased performance. Healthcare providers are the number one information source for rowers regarding exercise during pregnancy.

Conclusion

Pregnant rowers are a relevant obstetrics population and have barriers and sport-specific safety concerns not previously identified in the literature. Rowers consider exercising in pregnancy to be important and struggle to meet exercise guidelines like the general population, indicating the need for healthcare providers to provide prenatal and antenatal education and interventions to support exercise during pregnancy even amongst athletes.

## Introduction

The United States (US) Department of Health and Human Services encourages healthy pregnant and postpartum women to engage in at least 150 minutes of moderate-intensity aerobic activity per week [1], where moderate-intensity corresponds to a rating of perceived exertion (RPE) of 12-14 on the 15-grade Borg RPE Scale [2]. In the absence of medical complications or contraindications, regular exercise during pregnancy helps maintain or advance physical fitness, improves mental health, assists with weight management, and reduces the risk of gestational diabetes in obese women [3-7].

Pregnant athletes are a special population for consideration because they maintain training and competition during pregnancy and the postpartum period [8-9]. The American College of Obstetrics and Gynecology (ACOG) notes that pregnant athletes require “frequent and closer supervision” due to their involvement in strenuous training schedules throughout pregnancy, which may be continued in healthy pregnancies (p. 139) [10]. Although there is no definitive upper limit of safety, recent studies demonstrate abnormal changes in fetal heart rate, umbilical artery Doppler measurement, and uterine artery blood flow at intensities higher than 85% [11] and 90% [12] of maximum maternal heart rate (MHR).

Currently, rowing is not mentioned in guidelines despite its increasing popularity in the U.S., in addition to the existence of several considerations for pregnant rowers, including an increased theoretical risk for abdominal trauma. When a rower is unable to match boat speed, her oar may become caught by the water, a phenomenon known as “catching a crab,” which can result in the oar being forcefully reversed towards the face, trunk, and/or abdomen. Pregnant rowers must also be especially cognizant of environmental exposure, thermoregulation, and hydration, given that crew (rowing) is an outdoor water activity. Rowers experience extreme physiologic demands during training and competition [13], with physical endurance and power demands comparable to cross-country skiing, cycling, running, speed skating, and swimming [14]. Athletes expend anaerobic effort for 20-30% of a sprint race, and the proportion of anaerobic activity expenditure increases with shorter rowing distances [15].

**Figure 1:**
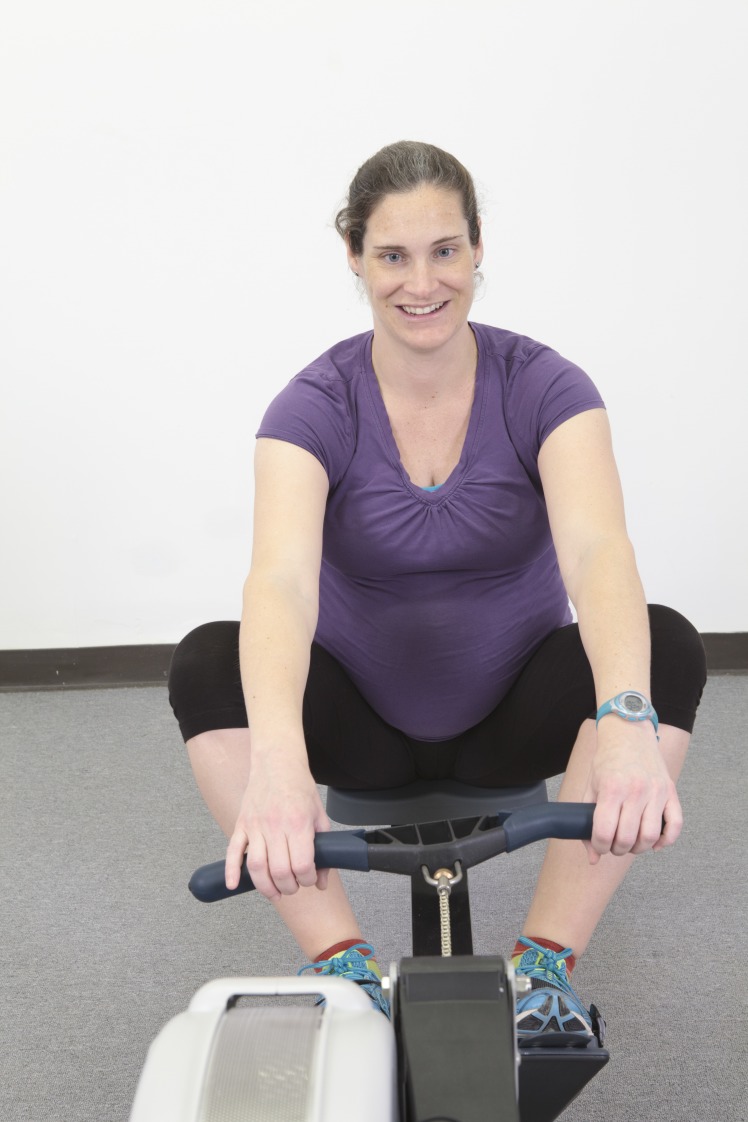
Pregnant Rower on Rowing Machine with Incorrect Form at the Catch Position Rower demonstrating how varus strain occurs when rowers try to accommodate the gravid abdomen to maintain their pre-pregnancy catch position. Image provided for use in this publication by Concept2, Inc. (http://www.concept2.com/)

Pregnant rowers are further specialized due to the uniqueness of the rowing stroke. The gravid abdomen restricts knee-to-chest compression necessary for a powerful stroke, placing physical limitations on the athletic ability that worsen throughout pregnancy and eventually prevent participation by most pregnant rowers in the sport (Figure 1). The primary aims of this research are to bring attention to the sport of rowing and to determine the existence of an athletic population engaging in competitive and recreational rowing during pregnancy. This study examines the self-reported behaviors, barriers to exercise, and safety concerns of rowers during pregnancy.

## Materials and methods

Participant recruitment

**Figure 2:**
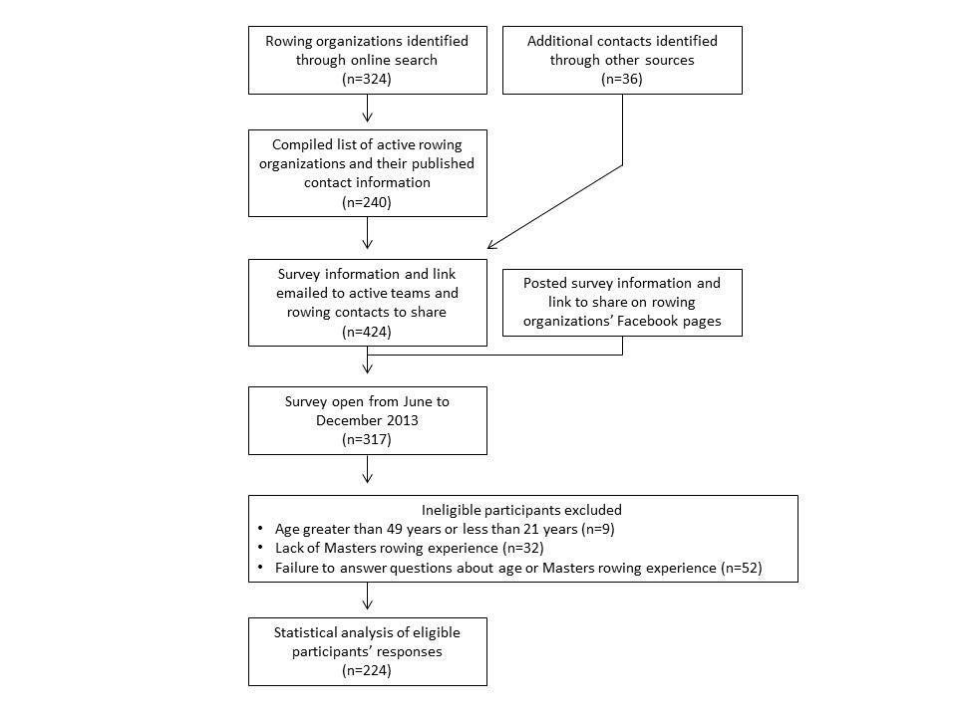
Study Flow Diagram

An invitation to share and/or complete a 122-item online survey was emailed to representatives of National and local Masters US rowing organizations and shared on various rowing and social media forums (Figure 2).

The email contained a link to a secure and confidential website where prospective participants could view a study explanation form and access the online survey, which remained open from June to December 2013. Inclusion criteria were female, Masters and/or Elite rower, and 21 to 49 years of age. Age minimum is defined by the definition of a Masters rower [16]. Age maximum is capped at 49 years, allowing women of childbearing age--identified by the Centers for Disease Control and Prevention as 21 to 44 years--the opportunity to report pregnancies within the past five years [17]. Our two initial hypotheses were as follows: (1) Rowers have unique safety concerns regarding exercise during pregnancy, and (2) pregnancy positively and/or negatively affects rowers' perceptions regarding exercise during pregnancy. As such, we chose to survey pregnant, non-pregnant, and never-pregnant rowers due to our interest in gaining all female rowers’ perspectives on rowing during pregnancy and/or with a pregnant teammate. The Institutional Review Board at the University of Central Florida approved this study prior to participant recruitment (approval #SBE-13-09282). All rowers provided informed consent for this study.

Data collection

Data were collected using a Qualtrics-generated survey (Qualtrics, Provo, UT) consisting of items in the following domains: (1) demographics; (2) rowing background; (3) obstetrical and gynecological history; (4) maternal beliefs about the barriers to physical activity during pregnancy; (5) maternal exercise information sources; (6) rowers’ beliefs regarding the importance of physical activity in pregnancy; (7) rowers’ beliefs regarding safety of moderate and vigorous exercise, erging, and rowing during pregnancy; and (8) rowers’ beliefs regarding the safety of competition during pregnancy. Domains 1 and 2 were modeled after the US Census Bureau categories and standard medical history questionnaires. Exercise is defined as, “Physical activity that is usually performed on a repeated basis over an extended period of time (exercise training) with a specific external objective, such as improvement of fitness, physical performance, or health” (p. 571) [18]. As such, “physical activity” and “exercise” will be used interchangeably. Note that rowing is a specific type of sport, exercise, physical activity, and competition. Therefore, athletes may identify as rowers but not compete, just like a runner may run but never race. See Table 1 for definitions of additional rowing- and study-specific terms.

**Table 1:** Definitions of Rowing- and Study-Specific Terms Definitions were directly quoted and/or created with reference to the 2016 United States Rowing Association (USRowing) Rules of Rowing and the International Olympic Committee's (IOC) expert group meeting on exercise and pregnancy in recreational and elite athletes.

2k split (“split”)	The amount of time in minutes it would take to row 500 meters at a given power.
2k power	A rower’s personal best time for erging 2,000 meters. Often used as a percentage (e.g., 50% of 2k power) with which to reference power output in a workout. This value can be measured in watts, which directly corresponds to a given 2k split.
Catch	The beginning of the rowing stroke when the oar blade enters the water. The position is characterized by shoulders flexed, arms extended, trunk flexion to ~30 degrees, knees flexed to 90 degrees and shoulder width apart, and maximum dorsiflexion.
Drive	The aspect of the rowing stroke in which the oar blade is propelled through the water.
Elite	“Competitor is Elite who has been a member of the Senior USRowing National Team or any country’s Senior National Team as a Competitor (including as a spare) or a medalist at the U23 World Championships in the category at issue.” [17]
Erg/Erging	The act of using an ergometer, also known as a rowing machine.
Exercise	“Physical activity that is usually performed on a repeated basis over an extended period of time (exercise training) with a specific external objective, such as improvement of fitness, physical performance, or health.” [17]
Finish	The end of the rowing stroke when the oar blade exits the water. The position is characterized by extended legs, back flexion of ~28 degrees, elbows bent, and shoulders abducted, internally rotated, and retracted.
Head race	“Race in which the participating Crews start a Race at different times, and where the order of finish is determined by comparison of the elapsed time taken to traverse the Course;” typically a distance of 5,000 meters.
Masters	“A Master is a Competitor who has attained or will attain the age of 21 during the current calendar year.” [16]
Rowing	“The propulsion of a displacement boat through water by the muscular force of one or more Rowers, with or without a Coxswain, in which oars are levers of the second order, and in which the Rowers are sitting with their backs to the direction of forward movement of the boat.” [16]
Scull	Refers to boats and events in which each rower uses two oars.
Sprint race	A rowing competition with a typical distance of 1,000 - 2,000 meters.

Barriers to exercise during pregnancy and maternal exercise information sources were assessed using a list of common responses modeled after Evenson, et al. [19] and Clarke, et al. [20]. We assessed the perceived importance of physical activity using a four-item scale taken from the study of Clarke, et al. [20]. Questions on rowers’ perceptions about the safety of activities during pregnancy (general exercise, erging, rowing, head race, and sprint race) were adapted using a scale taken from Mudd, et al. [21]. Definitions for moderate and vigorous exercise intensity were selected based on a lactate threshold (LT) training method developed by Concept2, Inc., Morrisville, VT, which relates the percentage of 2k power to percentages of heart rate reserve (HRR) [22]. Moderate intensity exercise is defined as “45 - 60% of 2k power; 70 - 80% of MHR” and vigorous intensity is defined as “greater than 90% of 2k power; greater than 90% of MHR” corresponding to a steady-state pace just below LT and to a pace in the anaerobic training zone, respectively. We selected 90% MHR specifically because of the Salvesen study [12]. Note that the term “vigorous” in this study is not the same as that defined by Borg and used in past studies [2]. The survey was validated using cognitive interviewing and debriefing with past collegiate and Masters rowers and coaches [23].

Statistical analysis

Participants who did not meet the inclusion criteria were removed from all analyses. Frequencies and percentages are reported for categorical variables. Sample sizes varied per item and are presented, where necessary, as survey items were not formatted in Qualtrics for required responses. When possible, participants were separated by gravidity and parity for analysis. Descriptive statistics for continuous variables are presented as the mean ± standard deviation (SD), with 95% confidence intervals (CI), or as median and inter-quartile range (IQR) for non-normal continuous variables and ordinal data (i.e., Likert-type rating scale items). The Friedman test was used to assess within-subjects differences across repeated measures items (i.e., first, second, third trimester safety item series). Follow-up pairwise comparisons using Wilcoxon signed ranks tests were conducted with Bonferroni correction to control for the inflation of family-wise error rate. Instrument reliability was assessed as internal consistency using Cronbach’s coefficient alpha. For data analysis of variables associated with the safety concerns of moderate and vigorous exercise, erging, and rowing during pregnancy, ratings of perceived safety were collapsed into safe (includes very safe and somewhat safe) and unsafe (includes very unsafe and somewhat unsafe), and ratings of unsure were not included for the purposes of conducting separate analyses between groups. Comparisons between groups of ordinal variables and non-normal continuous variables were conducted with non-parametric Mann-Whitney U tests, and between-group comparisons of normal continuous variables were conducted with independent samples t-tests. All tests were two-sided, and p-values < 0.05 for omnibus tests were considered statistically significant. Statistical analyses of quantitative data were completed with Statistical Package for Social Sciences (SPSS) 22.0 (IBM SPSS Statistics, Armonk, NY).

## Results

A total of 317 surveys were completed, and 224 surveys were available for analysis after excluding respondents who did not meet the inclusion criteria due to age (n = 9), lack of Masters rowing experience (n = 32), or a failure to answer questions regarding age, Masters rowing experience, or obstetrics history (n = 52). Response rate cannot be calculated since contacted research participants and rowing communities were asked to assist researchers in identifying other potential subjects through survey link sharing. Participant characteristics, including rowing experience, are presented in Table 2.

**Table 2:** Participant Characteristics Descriptive statistics are represented as indicated in the table with the N equal to 224, the total number of survey participants. Normal continuous data represented as mean and standard deviation (95% confidence interval). Non-normal continuous data presented as median (interquartile range; minimum-maximum). Absolute frequencies listed as n (%). One year of rowing equals participating in two rowing seasons. CI: confidence interval; SD: standard deviation

Characteristic	N = 224
	Mean ± SD (95% CI)
Age (years)	36.0 ± 7.6 (35.0 - 37.0)
	n (%)
Ethnicity	
American Indian or Alaskan Native	0 (0.0)
Asian	6 (2.7)
Black or African American	4 (1.8)
Native Hawaiian or Other Pacific Islander	1 (0.4)
Spanish or Hispanic or Latino	7 (3.1)
White	210 (93.8)
Marital Status	
Married	131 (58.5)
Domestic partnership	7 (3.1)
In a long-term relationship	33 (14.7)
Widowed	1 (0.4)
Divorced	7 (3.1)
Separated	1 (0.4)
Single	43 (19.2)
Highest Level of Education	
High school graduate or GED	1 (0.4)
Some college	6 (2.7)
Associate degree in college	3 (1.3)
Bachelor’s degree (For example: BA, AB, BS)	94 (42.0)
Master’s degree (For example: MA, MS, MEng, Med, MSW, MBA)	80 (35.7)
Doctorate degree or equivalent (For example: MD, PhD, DDS, DVM, LLB, JD)	40 (17.9)
Pregnant	18 (8.0)
	Median (25 percentile - 75 percentile; min-max)
Gravidity and Parity	1.0 (0.0-2.0; 0-10)
Rowing Experience	
Youth (years)	0 (0.0 - 1.0; 0 - 7)
Collegiate: NCAA Division I (years)	0 (0.0 - 1.0; 0 - 4)
Collegiate: NCAA Division II (years)	0 (0.0 - 0.0; 0 - 5)
Collegiate: NCAA Division III (years)	0 (0.0 - 0.0; 0 - 5)
Masters Rowing (years)	4.5 (2.3 - 9.0; 0 - 25)
	n (%)
National Rowing Team Experience	
Yes	18 (8.0)
Junior National	4 (1.8)
Under 23	12 (5.4)
Senior	8 (3.6)
Elite	8 (3.6)
No	205 (91.5)

Participants’ ages ranged from 21 to 49 years old with a mean of 36.0 ± 7.6 years. Masters rowing experience included a minimum value of 0.0 due to the inclusion of national team participants who were not yet recreational Masters. Eighteen of 224 women (8.0%) rowed at the National level. Gravidity ranged from 0.0 to 10.0 with a median of 1.0 pregnancy. Eighteen women (8.0%) were pregnant during the time of the survey. Intentions for future pregnancy were measured by the item, "Do you have a desire to become pregnant?"; answers ranged from "Yes, currently" (n = 13, 5.8%), "Yes, in the future" (n = 82, 36.6%), "No, Never" (n = 79, 35.3%), to "Unsure" (n = 28, 12.5%).

 The importance of exercise during pregnancy

A majority of surveyed women (N = 224) believe that it is important for a pregnant woman to exercise regularly (78.1%) and have an active lifestyle (88.8%). When asked about rowers’ familiarity with physical activity during pregnancy, 87.5% of women reported knowing someone who exercised during pregnancy and 66.1% reported knowing or hearing of someone who rowed during pregnancy.

Exercise patterns during pregnancy

Of the 115 rowers who indicated that they have had a past pregnancy, 98 women (85.2%) self-reported exercising for any amount of time in any past pregnancy. Descriptive data on exercise patterns are shown in Table 3.

**Table 3:** Exercise Patterns During Pregnancy by Trimester Descriptive statistics are presented as mean ± SD (95% CI), n. Frequencies were calculated using N = 115, the number of non-pregnant rowers with gravidity greater than 0, and are represented as n (%). Only 102 of 115 participants responded to the question, “Have you exercised for any amount of time during any past pregnancy?” CI: confidence interval; n: number; SD: standard deviation

	First Trimester	Second Trimester	Third Trimester
	Mean ± SD (95% CI), N		
Days per week	3.9 ± 1.5 (3.6 - 4.2), n = 94	3.8 ± 1.5 (3.5 - 4.1), n = 84	3.5 ± 1.6 (3.1 - 3.9), n = 77
Physical Activity Duration (minutes)	54.7 ± 22.9 (50.0 - 59.4), n= 93	50.9 ± 20.9 (46.4 - 55.4), n = 84	40.3 ± 16.9 (36.5 - 44.1), n = 78
Exercised 150 minutes or more per week	*n *(%)		
Very light	0 (0)	0 (0)	0 (0)
Light	3 (2.6)	2 (1.7)	13 (11.3)
Moderate	29 (25.2)	36 (31.3)	18 (15.7)
Vigorous	19 (16.5)	8 (7.0)	5 (4.3)
Very Hard	6 (5.2)	5 (4.3)	0 (0)
Maximum	5 (4.3)	0 (0)	0 (0)

Fewer than half of the rowers self-reported meeting exercise amount and intensity guidelines in the first (25.2%), second (31.3%), and third (15.7%) trimesters, respectively. A similar number of rowers exceeded guidelines by exercising at intensities of vigorous or higher for 150 minutes or more per week for the first (26.1%) and second trimesters (11.3%). A total of 32 women engaged in vigorous or higher intensity exercise of any duration in the first trimester per week. This number decreased through pregnancy with 15 women in the second trimester and six in the third engaging in higher intensity exercise. Thirty-six women (31.3%) competed in a regatta and 41 women (35.7%) competed in another sporting event. Only three of 18 women who were pregnant at the time of the study planned to compete in a regatta (16.7%) or in other athletic competitions during this pregnancy (16.7%).

Information sources for maternal exercise

The primary information sources for the pregnant rower were physician/midwife/other healthcare providers (60.2%), the internet (30.8%), and friend(s) (25.6%) (Table 4).

**Table 4:** Information Sources on Maternal Exercise Respondents asked, “What information sources did you use, if any, to decide how and whether or not you were/are going to exercise during this pregnancy?  (please select all that apply).” Results reported for the 133 surveyed rowers who indicated that they were pregnant at the time of survey or who have had a past pregnancy (gravidity > 0). Data are represented as absolute frequencies with N equal to 133. ACOG: American College of Obstetrics and Gynecology; ACSM: American College of Sports Medicine

Information source		Example Quote(s)
	*n* (%)	
Physician/Midwife/Other healthcare provider	80 (60.2)	
Internet	41 (30.8)	
Friend(s)	34 (25.6)	
Book or magazine	29 (21.8)	
Teammate/Exercise Partner	22 (16.5)	
Family/Spouse	18 (13.5)	
Other	10 (7.5)	
Personal monitoring	3 (2.3)	“Listened to my body,” “How I felt”
Peer-reviewed research	3 (2.3)	“ACOG” “ACSM” “IDEA Health & Fitness Assn. & Sara City Guidelines” “Research – I study exercise physiology”
Past experience	2 (1.5)	“Previous pregnancy”
Coach/Personal Trainer	5 (3.8)	
Did not seek information	1 (0.1)	

Women also cited personal monitoring (“listened to my body,” “how I felt”) as an exercise information source.

Barriers to exercise during pregnancy

The main barriers to physical activity during pregnancy included: (1) lack of energy, tired, sleepy (29.3%), (2) concerned for the baby (18.8%), and (3) nausea (14.3%) (Table 5).

**Table 5:** Rowers’ Main Barriers to Physical Activity During Pregnancy Respondents asked, “What are the three main reasons that kept you from being more active this pregnancy?” Answer list modified to include themes reported by Evenson [19] and Clarke [20], as well as a write-in option under the category, “other.” Results reported only for the 133 rowers who indicated that they were pregnant at the time of survey or who have had a past pregnancy (gravidity > 0). Data are represented as absolute frequencies, with N (number) equal to 133.

Barrier Categories	N = 133
No reason, I am physically active	46 (34.6)
Lack of energy, tired, sleepy	39 (29.3)
Concerned for the baby	25 (18.8)
Nausea	19 (14.3)
Concern with pregnancy complications	17 (12.8)
Exercise causes physical discomfort or pain	11 (8.3)
Caregiving duties	8 (6.0)
Told by physician not to exercise	8 (6.0)
Other	8 (6.0)

Rowers also wrote-in barriers not previously identified. Responses underwent thematic content analysis and are shown in Table 6.

**Table 6:** Rowers’ Novel Barriers to Physical Activity During Pregnancy Respondents asked, “What are the three main reasons that kept you from being more active this pregnancy?” Answer list modified to include themes reported by Evenson [19] and Clarke [20], as well as a write-in option under the category, “other.” Novel barriers identified through thematic content analysis of write-in responses are noted and example quotes included. Results reported only for the 133 rowers who indicated that they were pregnant at the time of survey or who have had a past pregnancy (gravidity > 0). Data are represented as absolute frequencies, with N (number) equal to 133.

Barrier Categories	N = 133	Example Quote(s)
Told by physician to avoid high-intensity exercises like rowing	2 (1.5)	“Told by physician to avoid rowing and strenuous training”
Guilt towards team	1 (0.8)	“Felt I was slowing down my teammates”
Mental barrier due to decreased performance	1 (0.8)	“Rowed until 6 months pregnant. I decided I was too slow, so I stopped rowing.”
Overly protective teammates	1 (0.8)	“Overly protective teammates!”
Physically unable to perform the rowing stroke	1 (0.8)	“Stomach muscles were the first thing to go and I was physically unable to even erg."
Trauma to abdomen	1 (0.8)	“I have a bad habit of jamming the oar handle into my stomach when I pull in or I would have rowed much further into pregnancy"
Unable to get into a boat	1 (0.8)	“Simply physically awkward getting in/out of rowing shell last 6 weeks of pregnancy “

Perceptions about safety when exercising during pregnancy

The primary reported rowing-specific safety concerns were: (1) abdominal trauma from the oar handle (37.9%) and physiological effects due to high intensities required by the sport (37.9%); (2) thermoregulation (8.0%); and (3) compression on and strain of the abdomen and pelvis at the catch, during the drive, and at the finish (6.3%). These and other novel concerns specific to pregnant rowers are shown in Table 7.

**Table 7:** Perceived Safety Concerns About Rowing During Pregnancy Respondents asked, "What aspect of rowing do you think is the main safety concern during pregnancy?" Safety concern categories and quotes obtained from analysis of write-in responses. Data are represented as absolute frequencies, with N (number) equal to the total number of survey participants, 224. Data are represented in absolute frequencies.

Safety Concerns		Example Quote(s)
	n (%)	
Abdominal trauma from the oar handle	85 (37.9%)	“catching a crab can cause the oar to hit your stomach” “finishing into the body too aggressively” “hitting your belly with an oar handle”
Physiological effects due to high intensities required by the sport	85 (37.9%)	“overexertion” “making sure heart rate does not go too high” “vigorous intensity requires too much oxygen” “anaerobic activity’s effect on the fetus” “increased adrenaline leading to stress response and possible miscarriage”
Thermoregulation	18 (8.0%)	“overheating” “risk of falling in cold water and having hypothermia”
Compression on and strain of the abdomen and pelvis at the catch, during the drive, and at the finish	14 (6.3%)	“getting to the catch could put too much pressure on your belly” “uterine pressure”
Musculoskeletal injury - due to changes in technique induced by a growing pregnancy	13 (5.8%)	“changing shape of body gets in the way of good technique”, “maintaining safe technique with expanding belly”
Flipping the boat	11 (4.9%)	“balance of the boat” “flipping while out rowing alone and being unable to pull self back in later in pregnancy”
Musculoskeletal injury - resulting from changes to the body during pregnancy	10 (4.5%)	“rowing is tough on the back, which is already in strain from the pregnancy” “the swing becomes difficult as abdominal muscles distend later in pregnancy”
Lack of available help when on the water	9 (4.0%)	“access to land and quickness of getting help” “being offshore”
Dehydration	6 (2.7%)	“dehydration”
Musculoskeletal injury	6 (2.7%)	
Lifting the boat in and out of the water	5 (2.2%)	“carrying a boat” “getting the boat from over-heads to down in the water without falling over”
An athlete's need to "push" themselves	3 (1.3%)	“temptation to exercise too vigorously while rowing with teammates and a good cox!”
Collision with another boat or object on the water	3 (1.3%)	“accidents in the boat or with other boats”
Drowning	3 (1.3%)	
Inability to moderate the intensity of the workout due to boat timing	3 (1.3%)	“in a team boat you don’t really have an option to stop or go at your own pace or the pace you feel is good for you”
Exposure to environmental elements	1 (0.4%)	“toxins in the water"
Getting in and out of the boat	1 (0.4%)	
Risk of infection from equipment	1 (0.4%)	

Median ratings of the perceived risk of participating in any of the specified physical activities during pregnancy increased significantly from first to third trimesters (p < 0.001); all follow-up pairwise comparisons between trimesters for each activity were also significant (all p < 0.001). Moderate intensity exercise of any activity during any trimester was perceived to be safer than vigorous intensity. General exercise was perceived to be the safest, whereas competing in a sprint or head race was felt to be most unsafe. Unsafe feelings toward a physical activity at a specified intensity and trimester were associated with a younger age, less rowing experience, and a gravidity of 0 (Tables 8-10). In general, rowers associated increased risk with on-water training and higher intensities.

**Table 8:** Correlation Analysis of the Perceived Safety of Physical Activities by Type, Intensity, and Trimester - First Trimester Data are expressed as mean ± standard deviation (SD) (95% confidence interval (CI)) for normal continuous variables, and as median (interquartile range; minimum-maximum) for non-normal continuous variables. P-values were derived from independent samples t-tests for normal continuous variables, and from Mann-Whitney U tests for non-normal continuous variables. Sample sizes (n) were consistent across age, masters rowing, and gravidity; however, n for living children delivered is presented separately, as these values reflect only those participants for whom parity > 0. n: number; p: probability value

Intensity Level	Activity	Belief	n	Age (years)	p	Masters Rowing (years)	p	Gravidity	p	n	Living Children Delivered	p
				Mean ± SD (95% CI)		Median (25th percentile-75th percentile; min-max)		Median (25th percentile-75th percentile; min-max)			Median (25th percentile-75th percentile; min-max)	
Moderate	Exercising	Safe	196	35.7 ± 7.6 (34.6 - 36.7)	0.44	4.5 (30.0 - 42.0; 0 - 25)	0.85	1.0 (0.0 - 2.0; 0 - 7)	0.49	116	1.0 (1.0 - 2.0; 0 - 4)	--
		Unsafe	2	31.5 ± 2.1 (12.4 - 50.6)		5.5 (30.0 - 33.0; 4 - 7)		0.5 (0.0 - 1.0; 0 - 1)		1	--	
	Erging	Safe	186	35.7 ± 7.6 (34.6 - 36.8)	0.74	4.8 (2.5 - 9.5; 0 - 25)	0.44	1.0 (0.0 - 2.0; 0 - 7)	0.23	108	1.0 (1.0 - 2.0; 0 - 4)	0.58
		Unsafe	5	34.6 ± 5.0 (28.4 - 40.8)		4.0 (1.0 - 7.0; 1 - 9)		2.0 (1.0 - 3.0; 0 - 3)		4	2.0 (1.0 - 2.0; 0 - 2)	
	Rowing	Safe	187	35.7 ± 7.5 (34.6 - 36.8)	0.91	4.5 (2.5 - 9.3; 0 - 25)	0.94	1.0 (0.0 - 2.0; 0 - 7)	0.51	110	1.0 (1.0 - 2.0; 0 - 4)	0.34
		Unsafe	6	35.3 ± 6.3 (28.7 - 41.9)		5.5 (3.0 - 9.0; 2 - 10)		1.5 (0.0 - 3.0; 0 - 3)		4	2.0 (1.0 - 2.5; 0 - 3)	
Vigorous	Exercising	Safe	130	35.9 ± 7.8 (34.5 - 37.2)	0.98	4.5 (2.5 - 9.0; 0 - 23)	0.53	1.0 (0.0 - 2.0; 0 - 5)	0.03	78	1.0 (1.0 - 2.0; 0 - 3)	0.46
		Unsafe	36	35.9 ± 6.6 (33.7 - 38.1)		4.8 (2.0 - 8.5; 0 - 25)		1.5 (0.0 - 3.0; 0 - 7)		26	1.5 (0.0 - 2.0; 0 - 4)	
	Erging	Safe	117	36.0 ± 7.5 (34.6 - 37.4)	0.77	5.0 (3.0 - 9.5; 0 - 23)	0.12	1.0 (0.0 - 2.0; 0 - 5)	0.10	73	1.0 (1.0 - 2.0; 0 - 3)	0.47
		Unsafe	38	35.6 ± 6.7 (33.4 - 37.8)		4.0 (1.5 - 8.0; 0 - 25)		1.0 (0.0 - 3.0; 0 - 7)		27	1.0 (1.0 - 2.0; 0 - 4)	
	Rowing	Safe	118	36.0 ± 7.5 (34.6 - 37.3)	0.55	5.0 (3.0 - 10.0; 0 - 23)	0.046	1.0 (0.0 - 2.0; 0 - 5)	0.22	74	1.0 (1.0 - 2.0; 0 - 3)	0.43
		Unsafe	40	35.2 ± 7.0 (32.9 - 37.4)		3.8 (1.3 - 7.5; 0 - 25)		1.0 (0.0 - 3.0; 0 - 7)		27	1.0 (1.0 - 2.0; 0 - 4)	
Competition	Sprint Race	Safe	97	36.5 ± 7.3 (35.1 - 38.0)	0.02	6.0 (3.0 - 10.5; 0 - 25)	0.01	1.0 (0.0 - 2.0; 0 - 7)	0.63	59	1.0 (1.0 - 2.0; 0 - 4)	0.73
		Unsafe	51	33.6 ± 6.8 (31.7 - 35.5)		3.5 (1.8 - 7.0; 0 - 23)		1.0 (0.0 - 2.0; 0 - 5)		31	1.0 (0.5 - 2.0; 0 - 4)	
	Head Race	Safe	116	36.6 ± 7.3 (35.2 - 37.9)	0.02	5.5 (2.8 - 10.8; 0 - 25)	0.09	1.0 (0.0 - 2.0; 0 - 7)	0.97	70	1.0 (1.0 - 2.0; 0 - 4)	0.58
		Unsafe	41	33.6 ± 6.9 (31.4 - 35.8)		4.0 (2.5 - 8.0; 0 - 23)		1.0 (0.0 - 2.0; 0 - 5)		25	1.0 (1.0 - 2.0; 0 - 4)	

**Table 9:** Correlation Analysis of the Perceived Safety of Physical Activities by Type, Intensity, and Trimester - Second Trimester Data are expressed as mean ± standard deviation (SD) (95% (CI) confidence interval) for normal continuous variables, and as median (interquartile range; minimum-maximum) for non-normal continuous variables. P-values were derived from independent samples t-tests for normal continuous variables, and from Mann-Whitney U tests for non-normal continuous variables. Sample sizes (n) were consistent across age, masters rowing, and gravidity; however,n for living children delivered is presented separately, as these values reflect only those participants for whom parity > 0. n: number; p: probability value

Intensity Level	Activity	Belief	n	Age (years)	p	Masters Rowing (years)	p	Gravidity	p	n	Living Children Delivered	p
				Mean ± SD (95% CI)		Median (25th percentile-75th percentile; min-max)		Median (25th percentile-75th percentile; min-max)			Median (25th percentile-75th percentile; min-max)	
Moderate	Exercising	Safe	187	35.8 ± 7.6 (34.7 - 36.9)	0.84	5.0 (2.5 - 9.3; 0 - 25)	0.83	1.0 (0.0 - 2.0; 0 - 7)	0.23	113	1.0 (1.0 - 2.0; 0 - 4)	--
		Unsafe	4	35.0 ± 4.8 (27.4 - 42.6)		5.8 (3.8 - 7.5; 3 - 8)		0.0 (0.0 - 1.0; 0 - 2)		1	--	
	Erging	Safe	173	35.9 ± 7.7 (34.8 - 37.1)	0.58	5.0 (2.5 - 10.0; 0 - 25)	0.25	1.0 (0.0 - 2.0; 0 - 7)	0.67	104	1.0 (1.0 - 2.0; 0 - 4)	0.93
		Unsafe	11	34.6 ± 5.2 (31.1 - 38.2)		4.0 (2.3 - 5.8; 1 - 9)		1.0 (0.0 - 2.0; 0 - 3)		6	1.5 (1.0 - 2.0; 0 - 2)	
	Rowing	Safe	170	36.3 ± 7.5 (35.1 - 37.4)	0.03	5.0 (2.5 - 10.0; 0 - 25)	0.06	1.0 (0.0 - 2.0; 0 - 7)	0.22	103	1.0 (1.0 - 2.0; 0 - 4)	0.99
		Unsafe	13	31.7 ± 6.4 (27.8-35.5)		3.0 (1.5 - 5.5; 1 - 9)		0.0 (0.0 - 1.0; 0 - 3)		6	1.0 (1.0 - 2.0; 1 - 2)	
Vigorous	Exercising	Safe	95	36.3 ± 7.7 (34.8 - 37.9)	0.06	5.0 (3.0 - 10.3; 0 - 23)	0.02	1.0 (0.0 - 2.0; 0 - 5)	0.71	57	1.0 (1.0 - 2.0; 0 - 3)	0.48
		Unsafe	60	34.0 ± 7.4 (32.1 - 35.9)		4.0 (1.5 - 8.0; 0 - 25)		1.0 (0.0 - 2.0; 0 - 7)		37	1.0 (0.0 - 2.0; 0 - 4)	
	Erging	Safe	82	36.8 ± 7.5 (35.2 - 38.5)	0.02	6.8 (3.5 - 11.0; (0 - 23)	0.003	1.0 (0.0 - 2.0; 0 - 5)	0.85	51	1.0 (1.0 - 2.0; 0 - 3)	0.27
		Unsafe	68	34.0 ± 7.5 (32.1 - 35.8)		3.8 (1.5 - 8.0; 0 - 25)		1.0 (0.0 - 2.0; 0 - 7)		41	1.0 (0.0 - 2.0; 0 - 4)	
	Rowing	Safe	79	36.9 ± 7.5 (35.3 - 38.6)	0.02	6.5 (3.3 - 10.8; 0 - 23)	0.01	1.0 (0.0 - 2.0; 0 - 5)	0.78	50	1.0 (1.0 - 2.0; 0 - 3)	0.33
		Unsafe	71	34.1 ± 7.6 (32.3 - 35.9)		4.0 (1.5 - 8.0; 0 - 25)		1.0 (0.0 - 2.0; 0 - 7)		43	1.0 (0.5 - 2.0; 0 - 4)	
Competition	Sprint Race	Safe	70	37.1 ± 7.1 (35.4 - 38.8)	0.003	5.3 (3.0 - 10.0; 0 - 25)	0.01	1.0 (0.0 - 2.0; 0 - 7)	0.40	45	1.0 (1.0 - 2.0; 0 - 4)	0.34
		Unsafe	80	33.6 ± 7.3 (31.9-35.2)		3.5 (1.5-7.0; 0-23)		1.0 (0.0 - 2.0; 0 - 5)		45	1.0 (0.0 - 2.0; 0 - 4)	
	Head Race	Safe	68	37.4 ± 7.1 (35.6 - 39.1)	0.01	6.8 (3.0 - 11.0; 0 - 22)	0.004	1.0 (0.0 - 2.0; 0 - 6)	0.37	45	1.0 (1.0 - 2.0; 0 - 3)	0.59
		Unsafe	74	33.9 ± 7.5 (32.2 - 35.7)		3.5 (1.5 - 7.0; 0 - 25)		1.0 (0.0 - 2.0; 0 - 7)		42	1.0 (0.0 - 2.0; 0 - 4)	

**Table 10:** Correlation Analysis of the Perceived Safety of Physical Activities by Type, Intensity, and Trimester - Third Trimester Data are expressed as mean ± standard deviation (SD) (95% (CI) confidence interval) for normal continuous variables, and as median (interquartile range; minimum-maximum) for non-normal continuous variables. P-values were derived from independent samples t-tests for normal continuous variables, and from Mann-Whitney U tests for non-normal continuous variables. Sample sizes (n) were consistent across age, masters rowing, and gravidity; however, n for living children delivered is presented separately, as these values reflect only those participants for whom parity > 0. n: number; p: probability value

Intensity Level	Activity	Belief	n	Age (years)	p	Masters Rowing (years)	p	Gravidity	p	n	Living Children Delivered	p
				Mean ± SD (95% CI)		Median (25th percentile-75th percentile; min-max)		Median (25th percentile-75th percentile; min-max)			Median (25th percentile-75th percentile; min-max)	
Moderate	Exercising	Safe	162	35.5 ± 7.5 (34.4 - 36.7)	0.95	4.8 (2.5 - 9.0; 0 - 23)	0.51	1.0 (0.0 - 2.0; 0 - 6)	0.22	97	1.0 (1.0 - 2.0; 0 - 4)	0.68
		Unsafe	15	35.4 ± 5.9 (32.1 - 38.7)		4.0 (2.8 - 7.0; 1 - 13)		0.0 (0.0 - 1.0; 0 - 4)		7	1.0 (1.0 - 1.0; 1 - 2)	
	Erging	Safe	134	36.0 ± 7.3 (34.8 - 37.3)	0.06	5.5 (2.5 - 10.0; 0 - 23)	0.01	1.0 (0.0 - 2.0; 0 - 6)	0.04	85	1.0 (1.0 - 2.0; 0 - 3)	0.69
		Unsafe	32	33.3 ± 6.8 (30.9 - 35.8)		3.3 (1.5 - 6.5; 0 - 13)		0.0 (0.0 - 1.0; 0 - 5)		15	1.0 (1.0 - 1.5; 0 - 4)	
	Rowing	Safe	118	36.7 ± 7.1 (35.4 - 38.0)	0.003	5.5 (3.0 - 10.0; 0 - 23)	0.01	1.0 (0.0 - 2.0; 0 - 6)	0.02	76	1.0 (1.0 - 2.0; 0 - 3)	0.89
		Unsafe	48	33.1 ± 7.3 (30.9 - 35.2)		3.3 (1.5 - 7.5; 0 - 18)		0.0 (0.0 - 1.0; 0 - 5)		23	1.0 (1.0 - 2.0; 0 - 4)	
Vigorous	Exercising	Safe	37	39.8 ± 7.5 (37.2 - 42.3)	< 0.001	7.5 (4.0 - 15.0; 0 - 23)	0.003	1.0 (0.0 - 2.0; 0 - 5)	0.44	25	1.0 (1.0 - 2.0; 0 - 3)	0.79
		Unsafe	99	34.3 ± 7.1 (32.9 - 35.8)		4.0 (2.0 - 8.0; 0 - 25)		1.0 (0.0 - 2.0; 0 - 7)		59	1.0 (1.0 - 2.0; 0 - 4)	
	Erging	Safe	29	39.5 ± 8.0 (36.5 - 42.5)	0.001	7.0 (3.0 - 13.5; 0 - 23)	0.04	1.0 (0.0 - 2.0; 0 - 5)	0.38	20	1.0 (1.0 - 2.0; 0 - 2)	0.83
		Unsafe	106	34.2 ± 7.2 (32.8 - 35.6)		4.0 (2.0 - 8.5; 0 - 25)		1.0 (0.0 - 2.0; 0 - 7)		62	1.0 (1.0 - 2.0; 0 - 4)	
	Rowing	Safe	28	39.8 ± 7.9 (36.7 - 42.8)	0.001	7.0 (3.0 - 11.5; 0 - 22)	0.12	1.5 (0.0 - 2.0; 0 - 5)	0.33	19	2.0 (1.0 - 2.0; 0 - 2)	0.53
		Unsafe	112	34.5 ± 7.3 (33.1 - 35.8)		4.0 (2.0 - 8.3; 0 - 25)		1.0 (0.0 - 2.0; 0 - 7)		66	1.0 (1.0 - 2.0; 0 - 4)	
Competition	Sprint Race	Safe	20	37.0 ± 7.6 (33.4 - 40.5)	0.25	4.5 (1.3 - 10.0; 0 - 22)	0.90	0.5 (0.0 - 2.0; 0 - 3)	0.42	10	2.0 (1.0 - 2.0; 0 - 3)	0.48
		Unsafe	123	34.9 ± 7.5 (33.5 - 36.2)		4.5 (2.0 - 9.0; 0 - 25)		1.0 (0.0 - 2.0; 0 - 7)		74	1.0 (1.0 - 2.0; 0 - 4)	
	Head Race	Safe	20	37.1 ± 7.1 (33.7 - 40.4)	0.26	5.5 (1.8 - 11.5; 0 - 23)	0.48	1.0 (0.0 - 2.0; 0 - 3)	0.77	12	1.0 (1.0 - 2.0; 0 - 3)	0.97
		Unsafe	118	35.0 ± 7.6 (33.6 - 36.4)		4.0 (2.0 - 9.0; 0 - 25)		1.0 (0.0 - 2.0; 0 - 7)		70	1.0 (1.0 - 2.0; 0 - 4)	

## Discussion

This is the first study to examine the self-reported behaviors, barriers to exercise, and safety concerns regarding exercise during pregnancy in a rowing population. Importantly, this research definitively demonstrates that a population of competitive pregnant rowers exists. 

Exercise patterns during pregnancy

This research demonstrates that rowers have higher levels of exercise adherence than the general population with 85.2% (n/N = 98/115) of rowers indicating that they exercised in a past pregnancy. Comparatively, a recent study reported rates of 12.7 to 45.0% of women exercising in pregnancy (n = 247), depending on the inclusion of lower physical activity thresholds, defined as greater than or equal to 100 minutes of physical activity [24]. Despite increased participation, only 51.3%, 42.4%, and 15.7% of pregnant rowers met and/or exceeded exercise guidelines (exercising at moderate intensity or higher for 150 minutes or more a week) in the first, second, and third trimesters, respectively, compared to the 17.0% of first-trimester, 14.1% of second-trimester, and 7.5% of third-trimester women in a general US study [25]. Comparing this paper's results to a study by Tenforde, et al. [8], our surveyed rowing population had higher rates of self-reported exercise engagement during pregnancy than their competitive running population. Rowers demonstrated a declining exercise trend from the first to third trimesters similar to past studies in the general population [26-28] and competitive athletes [8-9]. Table 11 summarizes the methods for past studies that examined exercise behavior in pregnancy.

**Table 11:** Studies Examining Exercise Behavior in Pregnant Women in the United States

General Population		
Hausenblas and Symons Downs [25]	2007	Longitudinal study assessing pregnant women’s exercise attitudes and behaviors Administered the Leisure Time Exercise Questionnaire to 89 pregnant women during the first, second, and third trimesters
Bordulin, et al. [26]	2008	Measured different modes, frequency, duration, and intensity of physical activity among 1,482 pregnant women Self-report via telephone interview of physical activity in past week at 17-22 and 27-30 weeks’ gestation
Daly, et al. [27]	2016	Observational study measuring physical activity and exercise behavior in 155 obese, post-partum women via questionnaire
Athletic Population		
Beilock, et al. [9]	2001	Observational study measuring training patterns before, during and after childbirth via survey of 26 competitive female athletes who had given birth within last 10 years
Tenforde, et al. [8]	2015	Observational, cross-sectional study measuring training attitudes and behaviors during pregnancy and postpartum among 110 female, long-distance runners via online survey

Such reduced rates of exercise adherence in the pregnant rowing athletic population emphasize the need to deliver prenatal and antenatal education and interventions to promote exercise during pregnancy even amongst athletes. Training regimens reported in this study suggest that some rowers perform high-intensity exercise (vigorous, very hard, and maximum intensity) during pregnancy that may potentially be unsafe when the duration of activity is considered based on the Syzmanski [11] and Salvesen [12] studies.

Barriers to exercise during pregnancy

This study provides evidence for the prevalence of some common conditions and complaints in pregnancy identified in Part 1 of the International Olympic Committee's (IOC) evidence summary that may interfere with exercise and competition during pregnancy [17]. The main self-reported barriers to exercise during pregnancy identified by rowers are nausea, concern for the baby, and lack of energy. Rowers also reported musculoskeletal complaints (i.e., diastasis recti abdominis) and reduced rowing range of motion due to the gravid abdomen. While the exact prevalence of these concerns was not measured, this study suggests that such barriers are significant enough to reduce exercise involvement during pregnancy. This could also explain the reduced rates of exercise engagement during pregnancy self-reported by rowers.

While this study demonstrates that rowers experience barriers to exercise in pregnancy similar to those in the general population, write-in responses reveal a unique perspective on the reasons why exercise adherence is difficult in pregnancy. Both the categories “guilt towards team” and “mental barrier due to decreased performance” demonstrate that some rowers are unwilling to engage in a particular exercise activity if they perceive a certain level of personal incompetence. This finding is very interesting because it indicates that rowers may make decisions regarding exercise during pregnancy that do not include concerns about their health or the health of their baby. Instead, rowers may make decisions about exercise in pregnancy based on concerns about their athletic identity. Follow-up interviews with survey participants and/or future research will be necessary to explore this idea further.

Rowers’ safety concerns for rowing during pregnancy

Rowers associated increased risk with on-water training, higher intensities, and increasing gestational age. Rowers in this study were significantly more likely to state that a physical activity at a specified intensity and trimester was unsafe if they were younger, had less rowing experience, or were nulliparous. This finding is consistent with the study by Mudd, et al. in which nulliparity was associated with feeling unsafe/unsure about vigorous physical activity [21]. Thus, rowers new to child-bearing may need more guidance and close follow-up during pregnancy in order to alleviate concerns and balance their reproductive and athletic goals.

Rowers reported many safety concerns for rowing during pregnancy, including those discussed in ACOG’s committee opinion on exercise in pregnancy [10]. We will thus discuss only those concerns that have not previously been mentioned in the literature.

Abdominal Trauma

While it is reasonable to consider the risks of abdominal trauma in rowing, there are no such cases in the literature. In addition, “catching a crab” is already a rare occurrence and has varying severity, so further research would need to be done to measure the actual risk that “catching a crab” confers to the developing fetus

Compression On and Strain of the Abdomen and Pelvis at the Catch, During the Drive, and at the Finish

Maximum knee-to-chest compression with maximum dorsiflexion and perpendicular shins is required to optimize rowing stroke power and length. While this action may be uncomfortable, if not impossible, depending on the size of a rower’s abdomen as well as their flexibility, the authors were primarily concerned about the risk of musculoskeletal injury secondary to technique changes made in an attempt to maintain adequate rowing length.

Musculoskeletal Injury - Due to Changes in Technique Induced by a Growing Pregnancy

Pregnant rowers who experience limitations on knee-to-chest compression by the gravid abdomen may try to compensate by externally rotating the hips, which would place significant varus knee strain. Rowers may make other changes to their technique to compensate for the physical changes of pregnancy. As such, it may be important for providers to encourage pregnant rowers to work with their coach and/or teammates to ensure proper technique and injury prevention.

Lack of Available Help When on the Water

This is a reasonable concern that may be easily mitigated by requesting that a coach or other individual accompany the pregnant rower through the use of a safety or coach's launch. Such boats are customary in rowing practices and required at rowing competitions.

Lifting the Boat In and Out of the Water

Providers may inform pregnant rowers of center of gravity changes in pregnancy, and encourage them to request help if they feel unsteady when launching a boat or returning from a row.

An Athlete's Need to "Push" Themselves

Rowers described their need to "push" themselves as a safety concern during pregnancy. Highly competitive pregnant athletes who are unable to suppress their drive to train may overexert themselves during pregnancy. Pivarnik, et al. noted a similar concern, warning that athletes “may have a no pain no gain mentality and may not listen to their bodies as well as they should” (p. 618) [28]. Interestingly, three rowers in this study reported the contrary, stating that personal monitoring served as their information source on maternal exercise. Ours is a similar finding to that of Fieril, et al. who reported four strategies in which women adapted exercise to pregnancy, one of which included being “extra attentive during exercise” (p. 1141) [29]. The authors noted that instant bodily feedback helped the woman know whether or not to modify and continue or stop the exercise. Therefore, we conclude that athletes have a strong understanding of their bodies, and thus, it is important for providers to be aware that pregnant athletes may at times be self-aware enough to modify exercise but may also be unable or unwilling to listen to physical warning signs during pregnancy.

Information sources regarding exercise during pregnancy

In this study, rowers cite healthcare providers as the primary information source regarding questions about exercise during pregnancy. Recent research on prenatal exercise counseling demonstrates that providers continue to recommend reducing exercise intensity and/or duration or advise pregnant women to maintain a heart rate below an arbitrary maternal heart rate (e.g., 140 bpm) based on old guidelines [28]. Providers also continue to cite a lack of sufficient knowledge regarding antepartum exercise guidelines [30]. Given our participants’ priority of seeking antenatal exercise advice from a healthcare provider, we emphasize the importance of including education on exercise during pregnancy in undergraduate and graduate medical education. While pregnant rowers primarily sought education from healthcare providers, rowers also reported asking a coach and/or teammate for advice. Healthcare providers of obstetric populations should, therefore, consider asking their pregnant athlete patients about any concerns or comments from the coach and/or team.

This study has some limitations. First, exercise intensity is defined differently than past studies. Second, it is possible that rowers under- or over-reported exercise intensity patterns during pregnancy. In addition, reliance on self-reporting presents a risk for recall bias.

## Conclusions

This study aimed to elicit information from competitive female rowers regarding exercise, training, and competition during pregnancy. We conclude that rowers consider exercising in pregnancy to be important and struggle to meet exercise guidelines like the general population. Our research definitively demonstrates that a population of competitive pregnant rowers exists, and that rowers have relevant, sport-specific safety concerns. Of note, pregnant rowers must be cognizant of the following: the effect of high exercise intensities; the risk of abdominal trauma; thermoregulation, hydration, and environmental exposure, given that rowing is performed outdoors on the water; and the physical limitations placed on athletic ability by the gravid abdomen that eventually prevents any participation by most rowers. The concerns of rowers presented in this study raise three practical questions for future investigation: (1) Is the theoretical concern for oar-induced abdominal trauma significant?; (2) Is it possible for rowers to make modifications to equipment to maximize rowing technique as the range of motion decreases?; and (3) If rowers make decisions regarding exercise during pregnancy based on concerns about their athletic identity, is there a dichotomy between pregnancy and athletics?; and if so, are rowers, and other female athletic populations, at risk of experiencing loss of athletic identity during pregnancy? To the best of our knowledge, this is the first study that examines rowers’ attitudes and self-reported behaviors regarding exercise during pregnancy. We believe that this work will guide providers on topics to be aware of when treating the pregnant rower, as well as serve as a framework for future studies on sport-specific concerns during pregnancy.
